# Nitrated Fatty-Acids Distribution in Storage Biomolecules during *Arabidopsis thaliana* Development

**DOI:** 10.3390/antiox11101869

**Published:** 2022-09-21

**Authors:** Lorena Aranda-Caño, Raquel Valderrama, Mounira Chaki, Juan C. Begara-Morales, Manuel Melguizo, Juan B. Barroso

**Affiliations:** 1 Group of Biochemistry and Cell Signaling in Nitric Oxide, Department of Experimental Biology, Faculty of Experimental Sciences, University Institute of Research in Olive Groves and Olive Oils, University of Jaén, E-23071 Jaén, Spain; 2 Department of Inorganic and Organic Chemistry, Faculty of Experimental Sciences, University of Jaén, E-23071 Jaén, Spain

**Keywords:** NO_2_-FAs, storage biomolecules, development, *Arabidopsis thaliana*, phospholipids, proteins

## Abstract

The non-enzymatic interaction of polyunsaturated fatty acids with nitric oxide (NO) and derived species results in the formation of nitrated fatty acids (NO_2_-FAs). These signaling molecules can release NO, reversibly esterify with complex lipids, and modulate protein function through the post-translational modification called nitroalkylation. To date, NO_2_-FAs act as signaling molecules during plant development in plant systems and are involved in defense responses against abiotic stress conditions. In this work, the previously unknown storage biomolecules of NO_2_-FAs in *Arabidopsis thaliana* were identified. In addition, the distribution of NO_2_-FAs in storage biomolecules during plant development was determined, with phytosterol esters (SE) and TAGs being reservoir biomolecules in seeds, which were replaced by phospholipids and proteins in the vegetative, generative, and senescence stages. The detected esterified NO_2_-FAs were nitro-linolenic acid (NO_2_-Ln), nitro-oleic acid (NO_2_-OA), and nitro-linoleic acid (NO_2_-LA). The last two were detected for the first time in Arabidopsis. The levels of the three NO_2_-FAs that were esterified in both lipid and protein storage biomolecules showed a decreasing pattern throughout Arabidopsis development. Esterification of NO_2_-FAs in phospholipids and proteins highlights their involvement in both biomembrane dynamics and signaling processes, respectively, during Arabidopsis plant development.

## 1. Introduction

The non-enzymatic interaction of polyunsaturated fatty acids with nitric oxide (NO) and derived species, such as dioxide of nitrogen (NO_2_) and peroxynitrite (ONOO^−^), gives rise to the formation of nitrated fatty acids (NO_2_-FAs), also known as nitrolipids or nitroalkenes [1]. Although the formation process of NO_2_-FAs in vivo is still unknown, two possible mechanisms have been proposed to explain the nitration of fatty acids. One consists of a direct radical–radical reaction between the ^·^NO_2_ radical and an alkyl radical. Nevertheless, this process has no biological relevance [2]. This is not the case of the second mechanism, which involves the formation of carbon-centered radicals by the direct addition of a NO_2_ radical. This radical can react with another NO_2_ radical to form an unstable nitro-nitrite or dinitro compound, which will rapidly decompose to release nitrous acid (HNO_2_) and generate a NO_2_-FA [1,3].

To date, the presence of nitro-oleic acid (NO_2_-OA), nitro-linoleic acid (NO_2_-LA), conjugated nitro-linoleic acid (NO_2_-cLA), and nitro-arachidonic acid (NO_2_-AA) has been identified in animal systems [4,5,6]. The presence of NO_2_-OA has been very recently detected in *Saccharomyces cerevisiae* [7]. In plant systems, the endogenous presence of NO_2_-cLA and cysteine-adducted NO_2_-OA has been identified in both olive fruit and extra virgin olive oil (EVOO) [8]. Additionally, nitro-linolenic acid (NO_2_-Ln) and NO_2_-OA have been detected in different plant species, such as *Arabidopsis thaliana* [9], *Pisum sativum*, *Oryza sativa* [10], and *Brassica napus* [11].

It is important to highlight that NO_2_-FAs can release NO through two possible mechanisms: the modified Nef reaction [12] and nitroalkene rearrangement [13,14]. However, the exact mechanism by which NO_2_-FAs release NO is not yet known. Several studies in *Arabidopsis thaliana* roots and cell cultures have shown that NO_2_-Ln is capable of releasing NO in vivo [15,16]. It has been recently described how the NO released by NO_2_-Ln can modulate nitrosogluthation (GSNO) levels in vitro and in vivo in Arabidopsis plants [17].

Lack of electrons in the β-carbon adjacent to the carbon (α-carbon) attached to the nitro group (-NO_2_) converts NO_2_-FAs into potent electrophiles. These molecules can establish covalent adducts with glutathione (GSH) and nucleophilic residues, such as cysteine, histidine, and lysine, by generating a post-translational modification (PTM) in proteins. This PTM is known as nitroalkylation, and it modifies protein structure and function [1,2,18,19,20]. Nitroalkylation is a reversible PTM that acts as a selective signaling pathway in stress situations. Under these conditions, increased reactive oxygen species (ROS) and reactive nitrogen species (RNS) levels can lead to the oxidation of the NO_2_-FA-protein bond (Michael adduct), which results in nitroalquene being released and the protein initial state being recovered [7,18,20].

NO_2_-FAs are generally important signaling molecules in animal, plant, and yeast systems. In animals, they have shown therapeutic benefits given their potent anti-inflammatory and cytoprotective effects in several experimental models [21,22,23,24,25,26]. In yeast, NO_2_-OA regulates the antioxidant response under heat stress by the nitroalkylation of peroxyredoxin Tsa1 [7]. In plants, they play a signaling role during plant development and under different abiotic stress conditions because NO_2_-Ln induces heat-shock transcription factors that regulate the expression of antioxidant systems [9], and NO_2_-OA triggers ROS production [27].

Moreover, NO_2_-FAs are considered stable and ubiquitous deposits of NO. This is because NO and its derived species have a very short half-life and perform their function in the vicinity of the place where they are generated. In an aqueous environment, if the concentration of NO_2_-FAs exceeds the critical micellar concentration (CMC), monomers transform into self-assembled structures, frequently micelles or reversible lipid aggregations, which have lower reactivity and greater stability [28]. Knowledge about the interactions between NO_2_-FAs and lipid aggregations is generally lacking, specifically for biomembranes. However by means of different computer simulation techniques, it has been shown that NO_2_-FAs can modify the permeability of cell membranes to form clusters at the membrane–water interface, which can in turn affect the dynamic structure of integral proteins [29].

In plant systems, biomembranes are composed mainly of phospholipids whose amphipathic character allows them to establish lipid bilayers. Phospholipids contain a glycerol molecule esterified with two fatty acids and a phosphate group to which different types of alcoholic groups (choline, ethanolamine, serine, inositol, etc.) can be attached. These lipid molecules are unevenly distributed among different cell membranes, abound more in extraplastidic membranes, and are scarcer in photosynthetic membranes [30,31]. Phosphatidylcholine (PC) and phosphatidylethanolamine (PE) are the most predominant phospholipids and perform a structural function [32,33]. Phosphatidylserine (PS) and phosphatidylinositol (PI) are less abundant but display key functions in signaling, trafficking, cell division, and growth in cell membranes [34,35].

Sphingolipids and phytosterol esters (SE) are also components of biomembranes but are barely present [36]. Sphingolipids are molecules generated by the union of a sphingoid base with a fatty acid [37]. This lipid class contributes to structural membrane integrity [38] and the formation of membrane domains [39,40], and it also participates in different signaling processes [41,42,43]. SE are the result of the esterification of phytosterols with fatty acids. The main phytosterols in Arabidopsis are β-sitosterol, stigmasterol, and campesterol [44]. This lipid class participates in membrane homeostasis maintenance [45].

Other plant lipid classes are triacylglycerides (TAGs), which result from the union of three fatty acids with a glycerol molecule. These biomolecules act mainly as a source of energy in early seed germination stages [46]. Mono- and diacylglycerides (MAGs and DAGs) are the common precursors of TAGs and phospholipids [47].

In short, proteins and complex lipids can be considered NO_2_-FAs storage biomolecules [19,28]. In animal systems, a set of proteins able to bind NO_2_-FAs has been reported [22,48,49,50,51,52,53,54,55,56]. In addition, TAGs have been identified as the main NO_2_-FAs storage biomolecule in these organisms [55]. However in plant systems, the NO_2_-FAs reservoir biomolecules and their biodistribution during Arabidopsis development are completely unknown and are the subject of this work. To date, only the drop in NO_2_-OA and NO_2_-Ln levels during the development of *Brassica napus* and *Arabidopsis thaliana*, respectively, has been described [9,11]. Therefore, the presence in *Arabidopsis thaliana* of not only NO_2_-Ln but also of NO_2_-OA and NO_2_-LA was identified in this paper. The storage biomolecules of each NO_2_-FA was also characterized, as was the distribution and NO_2_-FAs levels in storage biomolecules during *Arabidopsis thaliana* development. Finally, the possible role of NO_2_-FAs esterified with lipid storage and adducted with protein reservoirs was studied.

## 2. Materials and Methods

### 2.1. Plant Material

In this work, the plant species *A. thaliana* of the Columbia ecotype was used. The vegetal material from the dried seeds of different aged plants was used to characterize the development of this plant.

#### 2.1.1. Development Stages of *Arabidopsis thaliana*

The Bayer, BASF, Ciba-Geigy, and Hoechst (BBCH) scale, adapted to the Arabidopsis growth phenotypes, was used to select the development stages to be considered [57,58], and the plant vegetative, generative, and senescence stages were hence characterized. Specifically in the vegetative stage, the 5-, 14-, and 24-day-old plants were selected. From a phenotypical point of view, the 5-day-old seedlings showed completely open cotyledons, which represents the initial leaf development stage. In the 14-day-old seedlings, in addition to the two cotyledons, the circular arrangement of four leaves (rosette) was observed. This structure was completely configured in the 24-day-old plants, which presented a mature rosette constituted by a maximum of 13–14 leaves.

The 34- and 36-day-old plants represented the main events that took place in the generative stage. Particularly, the 34-day-old plants showed reproductive floral structures. Seed production was clearly observed inside the siliqua structures in the 36-day-old plants.

Finally, the senescence stage was represented by the 53-day-old plants, which phenotypically presented completely open, mature, yellow-brown siliques capable of releasing seeds.

The plant material used to characterize Arabidopsis development included the 5- and 14-day-old complete seedlings and the leaves from the 24-, 34-, 36-, and 53-day-old plants.

#### 2.1.2. Growing Conditions of *Arabidopsis thaliana*

*A. thaliana* seeds were sterilized for 10 min in 70% (*v*/*v*) ethanol solution containing 0.1% (*w*/*v*) sodium dodecyl sulfate (SDS). Seeds were then immersed for 20 min in sterile water containing 20% bleach (*v*/*v*) and 0.1% SDS (*w*/*v*). The next several washes were performed with sterile water, followed by a 5-minute incubation in a 1/200 algaecide dilution. Finally, they were washed 4 times with sterile water (5 min. each) and left at 4 °C for 2 days to break dormancy.

The Arabidopsis seedlings from the 5- and 14-day-old plants were grown in Petri plates according to the method described by [59]. In contrast, the 24-, 34-, 36-, and 53-day-old plants were obtained from hydroponic cultures under aeration using a specific growth medium [60]. Plants were grown under conditions of 16 h of light at 22 °C, 8 h of darkness at 18 °C, 60% humidity, and 100 μE m^–2^ s^–1^ light intensity. 

### 2.2. Reagents

MS medium for growing *Arabidopsis thaliana* plants, commercial oleic acid (OA), linoleic acid (LA), linolenic acid (Ln), and carbon 13-labelled OA (^13^C18-OA) as well as the different solvents herein used were purchased from Sigma-Aldrich (Saint Louis, MO, USA). The strata-NH_2_ solid-phase extraction columns were supplied by Phenomenex (Torrance, CA, USA).

### 2.3. Synthesis and Characterization of Standards NO_2_-OA, NO_2_-LA, and NO_2_-Ln and Internal Standard ^13^C18-NO_2_-OA by NMR Spectroscopy

The synthesis of standard NO_2_-OA was carried out by nitroselenation, oxidation, and hydroselenoxide elimination similarly to that previously described by [7]. Briefly, in a tetrahydrofuran–acetonitrile mixture (1:1, *v*/*v*, 24 mL), commercial OA (1 g, 3.05 mmol), solid mercury chloride (1.15 g, 4.23 mmol), phenylselenyl bromide (0.93 g, 3.93 mmol), and sodium nitrite (0.49 g, 7.19 mmol) were dissolved. This mixture was incubated in an argon atmosphere for 12 h. 

After incubation, the mixture was filtered and evaporated. The residue was dissolved in 12 mL of tetrahydrofuran, and after adding 33% (*v*/*v*) hydrogen peroxide (3.63 mL), the mixture was stirred for 1 h in an ice bath. This step was followed by extraction with hexane. The solvent was then washed with saturated sodium chloride solution, and any water was removed with anhydrous magnesium sulfate. Next, the sample was evaporated. NO_2_-OA purification was performed by flash column chromatography (silica gel 60) with a mixture of hexane/diethyl ether/acetic acid (80:20:0.5, *v*/*v*/*v*) as the mobile phase. The selection of the fractions of interest was carried out on TLC plates (Sigma-Aldrich, Saint Louis, MO, USA) and developed with the same solvent mixture used in the previous chromatography. Finally, the structure of the synthesized compound was analyzed by NMR using a Bruker Avance 400 spectrometer (Billerica, MA, USA) that operated at 400.13 MHz for 1H and 100.61 MHz for ^13^C. 

Carbon 13-labelled NO_2_-OA (^13^C18-NO_2_-OA), which was used as an internal standard in the NO_2_-FAs quantification protocol, was synthesized as described above.

For the synthesis of standards NO_2_-LA and NO_2_-Ln, the above protocol was also followed, and only the incubation period of the nitroselenation process was changed, which was shortened to 4 h in an argon atmosphere instead of 12 h.

### 2.4. Detection of NO_2_-FAs from Lipid Storages

#### 2.4.1. Lipid Extraction

The plant samples that came from the different developmental stages were homogenized with liquid nitrogen to achieve greater cell wall and tissue disintegration. The lipid material was then extracted by the Bligh and Dyer technique using a mixture of hexane/isopropanol/1M formic acid (30:20:2, *v*/*v*/*v*) [61] before performing a solid-phase extraction. 

#### 2.4.2. Solid-Phase Extraction

The lipid material obtained in the Section 2.4.1. was chromatographically separated following the protocol described by [55] with a few modifications. This protocol allows the individual separation and collection of each of the fractions according to their polarity using solvents of increasing polarity. For this purpose, Strata-NH_2_ solid-phase extraction (SPE) columns were used, and SE, TAGs, MAGs-DAGs, free fatty acids (FFA), PC, PE, PS, PS, and PI were sequentially eluted with 12 mL of hexane, hexane/chloroform/ethyl acetate (100:5:5, *v*/*v*/*v*), chloroform/isopropanol (2:1, *v*/*v*), diethyl ether/2% acetic acid, acetonitrile/1-propanol (2:1, *v*/*v*), methanol, isopropanol/methanolic HCl (4:1, *v*/*v*), and methanol/methanolic HCl (9:1, *v*/*v*), respectively. The chromatographic fractions were then evaporated and later resuspended in 50 µL methanol, and 10 nM ^13^C_18_-NO_2_-OA was added as internal standard.

#### 2.4.3. Identification and Confirmation of Complex Lipid Class

The detection and confirmation of the different complex lipids obtained after the chromatography with Strata-NH_2_ columns was carried out following the protocol described by Baker et al. [62] by direct infusion MS/MS spectrometry. For this purpose, the all fractions were solubilized in 100 μL of a 300 mM methanol/chloroform/ammonium acetate mixture (665:300:35, *v*/*v*/*v*) with the exception of PS, to which 8 μL of acetic acid was also added. MS/MS analysis was carried out on an Agilent QTOF mass spectrometer sending the samples directly to the ion source (operating in positive mode). All lipids were measured using the following equipment settings: drying gas flow: 4 l/min nitrogen; fragmentation voltage: 100 V; gas temperature: 325 °C; scan rate: 1 spectra/s. 

To identify each lipid class, a representative molecular specie of each type of complex lipid, for which we knew the molecular adduct ion [M + H]^+^ or [M + NH_4_]^+^ and the molecular mass corresponding to the adduct ion (*m*/*z*), were used as a standard. Fragmentation of TAGs and DAGs was characterized by the neutral loss of the adduct corresponding to the fatty acid + NH_3_, which could be detected by a neutral loss scan for the indicated mass. Fragmentation of phospholipids, with the exception of PC, was also detected by scanning for the difference of the neutral loss corresponding to the adduct formed by the phospholipid head group. In the case of PC, detection was carried out by scanning the precursor ion corresponding to its head group.

#### 2.4.4. Acid Hydrolysis

In order to proceed to the detection and quantification of NO_2_-FAs from the lipid storages, the chromatographic fractions obtained following the protocol described in Section 2.4.2, SE, TAG, MAG-DAG, PC, PE, PS, and PI, with the exception of the FFA fraction, were subjected to acid hydrolysis to separate NO_2_-FAs from their corresponding complex lipid. To limit artificial nitration reactions catalyzed by the subsequent acid hydrolysis process, 250 µL of methanolic sulfanilamide (1 g/10 mL) were added to all fractions and incubated for 20 min. Next, the sample was evaporated and incubated in 2.5 mL of acetonitrile/hydrochloric acid (9:1, *v*/*v*) for 1 h at 90 °C. After acid hydrolysis, NO_2_-FAs were extracted with hexane/H_2_O (2:1, *v*/*v*). Finally, the hexane fraction was isolated, evaporated, and resuspended in methanol to be analyzed by LC-MS/MS.

### 2.5. Detection of NO_2_-FAs from Protein Storage

The plant sample was homogenized in liquid nitrogen with a specific buffer at the 1:2 (*w*/*v*) ratio (0.1 M Tris-HCl, pH 7.6, 7% sucrose (*w*/*v*); 7% PVPP (*w*/*v*); 0.05% Triton X-100 (*v*/*v*) and 0.1 mM EDTA). The sample was then centrifuged twice at 10,000 xg for 15 min at 4 °C. The obtained supernatant was precipitated with 70% (*v*/*v*) cold acetone (−20 °C) and stored overnight at −20 °C. After this time, the sample was recentrifuged at 10,000× *g* for 15 min at 4 °C.

The protein extracts obtained from acetone precipitation were dissolved in 2 mL of methanol. Next, the oxidation of the NO_2_-FAs-proteins adducts was carried out following the method described by [20] with some modifications. Firstly, the sample was divided into two equal parts. One part was non-oxidized and used to quantify the free NO_2_-FAs levels that could result from the acetone precipitation step. The other part of the sample was incubated with 50 mM of H_2_O_2_, which were added under shaking the infusion every 20 min for 3 h and 20 min. The treatment with H_2_O_2_ oxidizes Michael adducts with the subsequent NO_2_-FAs release.

A known concentration of the internal standard, ^13^C18-NO_2_-OA (10 nM), was added to both the non-oxidized and oxidized samples. Next, the FFA were extracted by adding 7.5 mL of hexane and vigorously shaking for 2 min. To properly separate phases, the mixture was centrifuged at 1000× *g* for 1 min at 4 °C. The upper apolar phase corresponding to hexane, rich in fatty acids, was collected, evaporated, resuspended in methanol, and analyzed by LC-MS/MS.

### 2.6. Detection and Identification of NO_2_-FAs by LC-MS/MS

Characterization and quantification of NO_2_-FAs obtained from both the FFA fraction and the complex lipid fractions after acid hydrolysis were performed following the protocol described in [7] with some modifications. A Sciex Triple Quadrupole Linear Trap Mass Spectrometer (QTRAP 6500+), coupled to a Sciex ExionLC AD ultrahigh-performance liquid chromatograph (UHPLC), was used. Lipid extracts were separated using a Kinetex 1.7 μm C18 100 Å (150 mm × 2.1 mm) column (Phenomenex, Torrance, CA, USA) in a solvent system consisting of water with 0.1% formic acid (A) and methanol with 0.1% formic acid (B).

The MS/MS analysis was performed in the negative ion mode. The presence of the different NO2-FAs was detected by applying the multiple reaction monitoring (MRM) scanning mode with the specific MRM transitions corresponding to NO_2_-OA (326/46 and 326/279 *m*/*z*), ^13^C18-NO_2_-OA (344.1/46 *m*/*z*), NO_2_-LA (324/46 and 324/277 *m*/*z*), and NO_2_-Ln (322/46 and 322/275 *m*/*z*) [63,64].

The quantification of the NO_2_-FAs levels was performed using a calibration curve prepared with the reference standard for each NO_2_-FA and with the presence of ^13^C18-NO_2_-OA as the internal standard. In all these cases, the obtained data were analyzed and processed with the Sciex OS software version 1.7.0.36606 (Ab Sciex, Redwood City, CA, USA).

### 2.7. Statistical Analysis

Statistical procedures were carried out by the Student’s *t*-test. Values represent the mean ± SEM of at least three biological replicates with significance when at least *p* < 0.05.

## 3. Results

### 3.1. Identification of the Main Endogenous NO_2_-FAs in Arabidopsis thaliana

NO_2_-FAs such as NO_2_-OA and NO_2_-LA play an important signaling role in animal systems [48,51,65]. However, in plant systems, only the presence of NO_2_-OA and NO_2_-Ln has been identified during the development of *Brassica napus* [11] and *Arabidopsis thaliana* [9], respectively. Additionally, the participation of NO_2_-Ln in the response to different stress situations has been described [9]. Given the relevance of NO_2_-OA, NO_2_-LA, and NO_2_-Ln in both animal and plant systems, this work applied mass spectrometry techniques and detected the endogenous presence of NO_2_-OA and NO_2_-LA in 14-day-old *Arabidopsis thaliana* seedlings for the first time. The existence of NO_2_-Ln was also corroborated. The 14-day-old seedlings were employed as a representative example of Arabidopsis plants because they are juvenile organisms in which flowering and gametogenesis processes have not yet occurred. 

For this purpose, MRM transitions corresponding to NO_2_-OA (*m*/*z* 326/46), NO_2_-LA (*m*/*z* 324/46), and NO_2_-Ln (*m*/*z* 322/46) were monitored. Otherwise, *m*/*z* 326, 324, and 322 were the main ion products that corresponded to the complete ion mass spectra (MS) of standards NO_2_-OA, NO_2_-LA, and NO_2_-Ln, respectively, when analyzed in the negative ion mode. In contrast, the MS/MS (MS2) spectra showed a major fragment with *m*/*z* 46 corresponding to the NO_2_ molecule.

**Figure 1:**
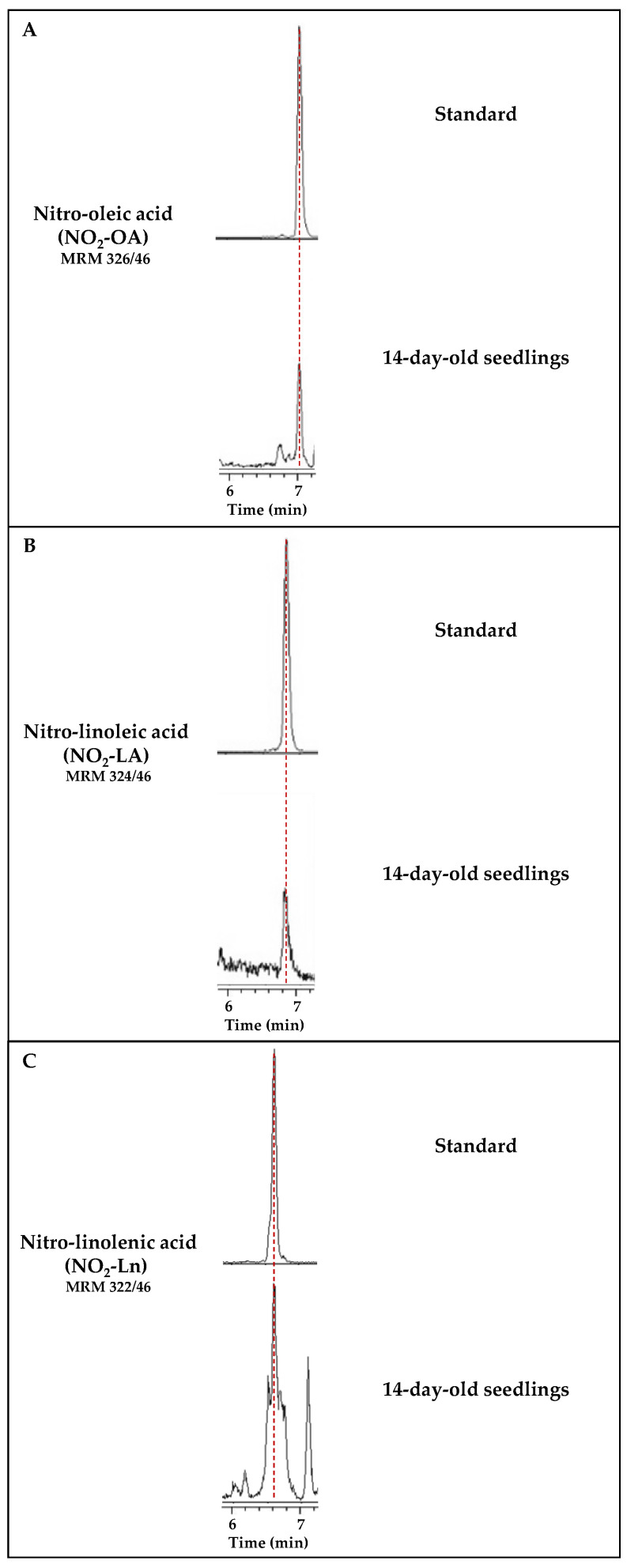
Detection of the main NO_2_-FAs present in *Arabidopsis thaliana*. Each panel shows the chromatogram corresponding to the standard of each nitrated fatty acid, NO_2_-OA (**A**), NO_2_-LA (**B**), and NO_2_-Ln (**C**). Their retention times coincide with the chromatographic peaks detected in the Arabidopsis lipid extracts from the 14-day-old seedlings. Peaks refer to a total ion intensity of 1.2 × 10^4^ for the *m*/*z* transition of NO_2_-OA (326/46), 4 × 10^3^ for NO_2_-LA (*m*/*z* 324/46), and 1.4 × 10^4^ for NO_2_-Ln (*m*/*z* 322/46). The dotted vertical line indicates the peaks with the same retention time.

Figure 1 shows the chromatographic peaks for the MRM transitions of standards NO_2_-FAs and the 14-day-old seedlings. In this case, the sample showed a chromatographic peak coincident with the MRM 326/46 transition, which shared the same retention time as standard NO_2_-OA (Figure 1A). The same behavior was observed for the MRM 324/46 transition corresponding to NO_2_-LA (Figure 1B). In this work, the presence of NO_2_-Ln, already published in Arabidopsis [9], was also corroborated (Figure 1C). 

**Figure 2:**
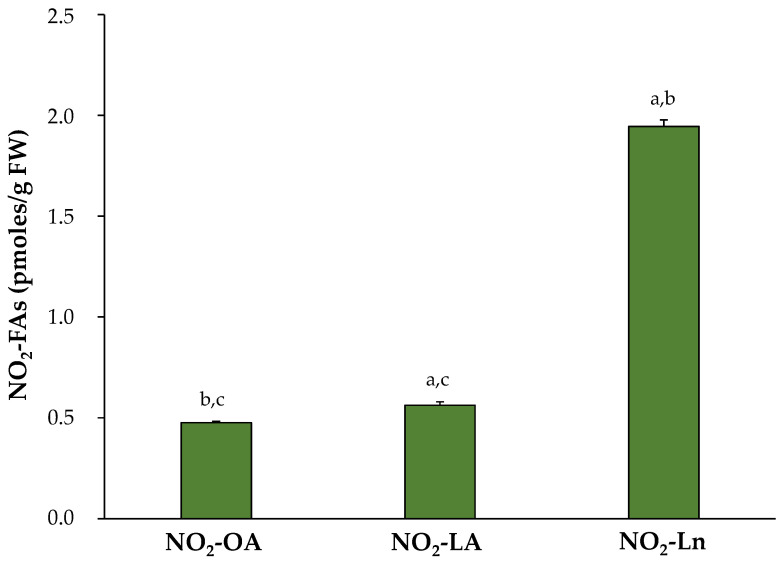
Endogenous NO_2_-FAs levels in the 14-day-old *Arabidopsis thaliana* seedlings. The NO_2_-FAs values (pmoles/g FW) are those obtained from the mean ± SEM of at least ten independent experiments. The statistical significance between means was analyzed by the Student’s *t*-test. Letters a, b, and c denote significant differences (*p* < 0.05) with NO_2_-OA, NO_2_-LA, and NO_2_-Ln, respectively. FW, fresh weight.

After identifying and confirming the presence of the three NO_2_-FAs in Arabidopsis, their levels were quantified in the 14-day-old seedlings. The most abundant NO_2_-FA was NO_2_-Ln (1.945 ± 0.032 pmol/g FW), followed by NO_2_-LA (0.563 ± 0.016 pmol/g FW) and NO_2_-OA (0.476 ± 0.007 pmol/g FW) (Figure 2).

### 3.2. Characterization of the Main NO_2_-FAs Storage Biomolecules in Arabidopsis thaliana

The physico-chemical properties of NO_2_-FAs allow their esterification with proteins and complex lipids, molecules that thus become NO_2_-FAs storage biomolecules. In animal systems, the esterification of NO_2_-FAs to complex lipids has been reported, where TAGs are the main reservoir [54,56]. In parallel, different proteins susceptible to bind NO_2_-FAs have also been characterized in animals [22,48,49,50,51,52,53,54,55,56]. However, in plant systems, all these aspects are completely unknown and constitute the purpose of this work.

The identification of NO_2_-FAs storage biomolecules was carried out in the 14-day-old Arabidopsis seedlings, which are a representative example of vegetative Arabidopsis growth. The characterization of the storage of NO_2_-FAs in lipid biomolecules was performed by modifying a chromatographic method previously used in animal systems [54]. This method allows complex lipids to be separated into different fractions according to their polarity, such as SE, TAGs, MAGs-DAGs, PC, PE, PS, and PI. We developed a new methodology for the detection of NO_2_-FAs from the adduction of proteins to look into the protein reservoir.

As shown in Supplementary Figure S1, all three NO_2_-FAs are present in both the protein and lipid storages. A detailed analysis of the NO_2_-FAs distribution in the storage biomolecules showed that in SE, NO_2_-Ln was the most abundant (0.064 ± 0.007 pmol/g FW), followed by NO_2_-LA (0.039 ± 0.007 pmol/g FW) and NO_2_-OA (0.029 ± 0.005 pmol/g FW) (Figure S1A). In TAGs, the three NO_2_-FAs were present at similar levels: 0.04 ± 0.007 pmol/g FW for NO_2_-OA, 0.035 ± 0.008 pmol/g FW for NO_2_-LA, and 0.032 ± 0.003 pmol/g FW for NO_2_-Ln (Figure S1B). In MAGs-DAGs storage, the amounts of both NO_2_-Ln (0.099 ± 0.011 pmol/g FW) and NO_2_-OA (0.067 ± 0.012 pmol/g FW) were considerable, but NO_2_-LA was less represented (0.022 ± 0.003 pmol/g FW) (Figure S1C). Abundance patterns differed for phospholipids. In PC, the presence of NO_2_-OA (0.057 ± 0.009 pmol/g FW) predominated, followed by NO_2_-Ln (0.03 ± 0.006 pmol/g FW) and NO_2_-LA (0.016 ± 0.002 pmol/g FW) (Figure S1D). In PE, NO_2_-OA (0.045 ± 0.007 pmol/g FW) and NO_2_-Ln (0.05 ± 0.005 pmol/g FW) showed similar quantities, while the participation of NO_2_-LA (0.011 ± 0.002 pmol/g FW) was lesser (Figure S1E). NO_2_-Ln (1.453 ± 0.18 pmol/g FW) was located mainly in the PS reservoir biomolecule where the other two NO_2_-FAs were the minority (NO_2_-LA: 0.127 ± 0.024 pmol/g FW; NO_2_-OA: 0.059 ± 0.009 pmoles/g FW) (Figure S1F). Finally, the levels of the three NO_2_-FAs in PI showed the same abundance pattern as that described for PE; that is, 0.062 ± 0.007 pmoles/g FW for NO_2_-OA, 0.053 ± 0.007 pmoles/g FW for NO_2_-Ln, and 0.026 ± 0.006 pmoles/g FW for NO_2_-LA (Figure S1G).

Proteins are other NO_2_-FAs storage biomolecules to consider. We developed a new methodology that allowed us to break the nitroalkylation adduct between NO_2_-FAs and proteins. In the 14-day-old seedlings, we detected similar levels for the three NO_2_-FAs: 0.1 ± 0.01 pmol/g FW for NO_2_-OA, 0.15 ± 0.02 pmol/g FW for NO_2_-LA, and 0.13 ± 0.02 pmol/g FW for NO_2_-Ln (Figure S1H). Finally, it should be noted that a small amount of each NO_2_-FA was neither adducted with proteins nor esterified with complex lipids, which constitutes the free nitrated fatty acids fraction (FFA) (Figure S1I). 

**Figure 3:**
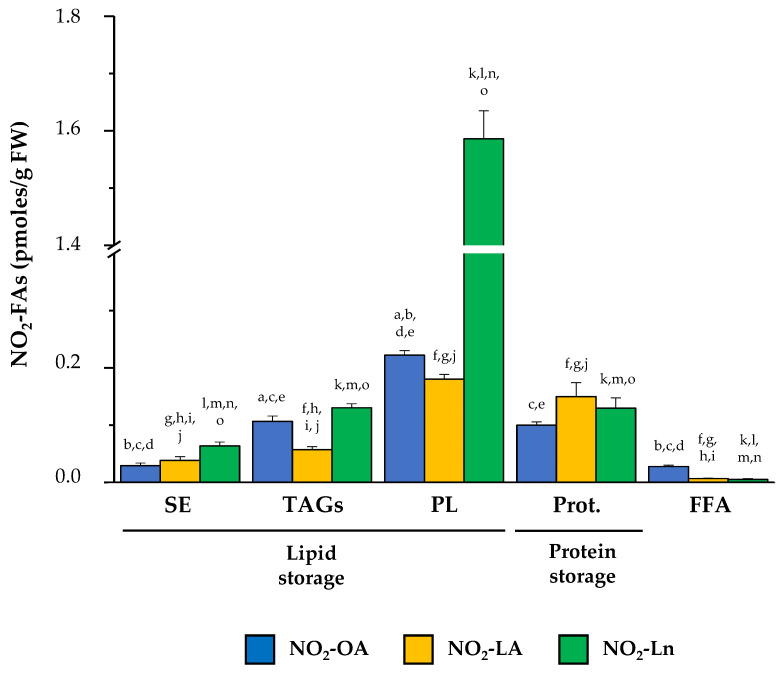
The main NO_2_-FAs storage biomolecules in *Arabidopsis thaliana*. This figure shows the different lipid and protein reservoirs of NO_2_-FAs in the 14-day-old Arabidopsis seedlings. The lipid deposit consisted of SE, TAGs (including MAGs-DAGs), and the four phospholipid types (PC, PE, PS, and PI). The protein deposit is represented by the NO_2_-FAs that were experimentally released from adducts with proteins. The FFA levels, which were neither esterified nor adducted, are also shown. The NO_2_-FAs values are the mean ± SEM of at least ten independent experiments. The statistical significance between means was analyzed by the Student’s *t*-test. Letters a, b, c, d, and e indicate the significant differences (*p* < 0.05) between the NO_2_-OA levels found in SE (a), TAGs (b), PL (c), Prot. (d), and FFA (e). Letters f, g, h, i, and j denote the significant differences (*p* < 0.05) between the NO_2_-LA levels found in SE (f), TAGs (g), PL (h), Prot. (i), and FFA (j). Letters k, l, m, n, and o represent the significant differences (*p* < 0.05) between the NO_2_-Ln levels found in SE (k), TAGs (l), PL (m), Prot. (n), and FFA (o). PL, phospholipids; Prot., proteins.

After combining the information obtained in our experiments about the distribution of NO_2_-FAs in both biological reservoir types (Figure 3), we conclude that the lipid reservoir, mainly represented by phospholipids, can be considered the main reservoir of NO_2_-OA and NO_2_-Ln. With NO_2_-LA, proteins and phospholipids appeared as the main storage biomolecules because NO_2_-LA presented similar levels in both reservoirs. The three NO_2_-FAs showed a different distribution in the storage biomolecules. The NO_2_-Ln reservoirs that were ordered from greater to lesser abundance were phospholipids, proteins together with TAGs, and finally SE. For NO_2_-LA, both phospholipids and proteins were preferential reservoirs, followed by TAGs and SE. Finally, the participation of NO_2_-OA in phospholipids was greater than in TAGs and proteins, while SE appeared as a minority deposit. The NO_2_-FAs levels in their free form were practically undetectable (Figure 3).

### 3.3. Endogenous Levels of NO_2_-FAs during Arabidopsis thaliana Development

**Figure 4:**
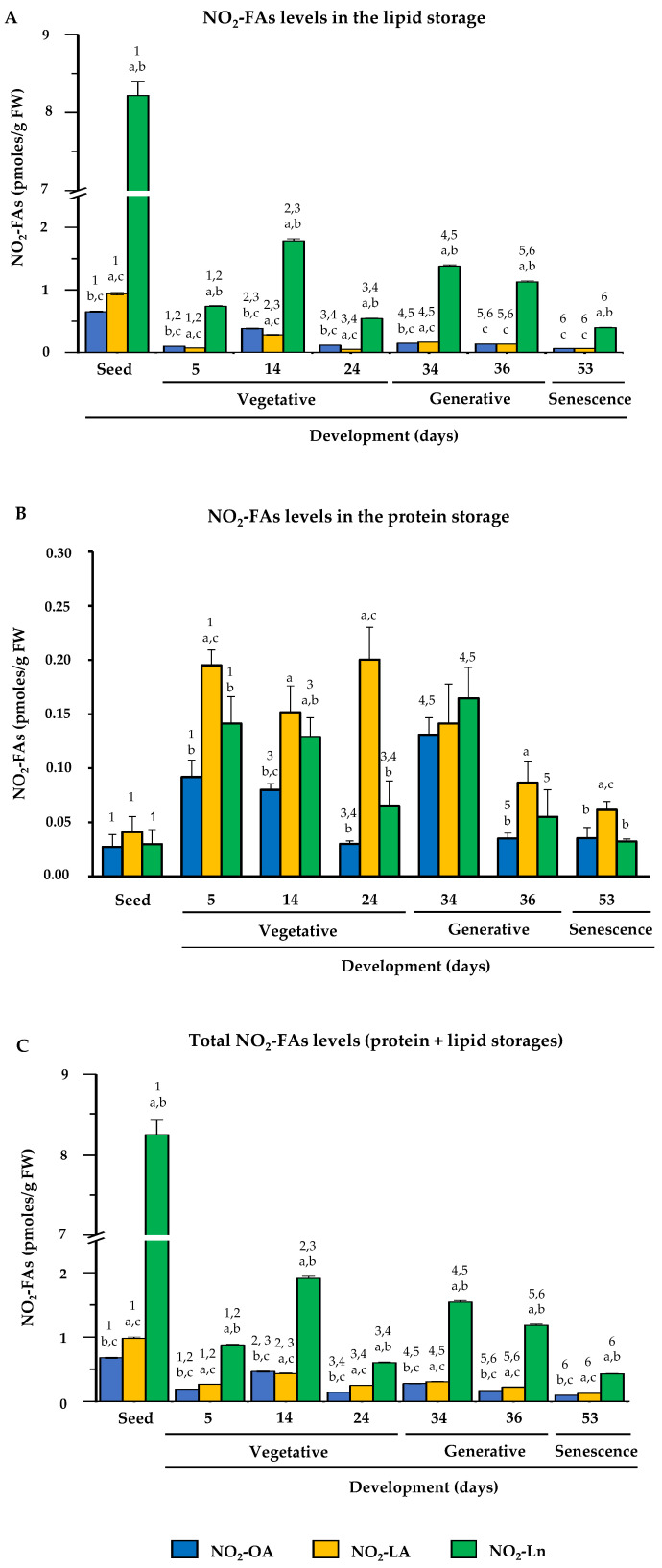
NO_2_-FAs endogenous levels during Arabidopsis development. This figure shows the quantification of the NO_2_-OA, NO_2_-LA, and NO_2_-Ln contents in the different plant development stages, which ranged from seed and vegetative stages (seed germination (at 5 days) and initial and complete rosette development (at 14 and 24 days, respectively)), generative stage (flowering (at 34 days), and seed production (at 36 days)) and senescence stage (at 53 days). Panel (**A**) displays the NO_2_-FAs endogenous levels esterified with the lipid storage biomolecules. Panel (**B**) shows the levels of the NO_2_-FAs from the adduction with the protein reservoir biomolecules. Panel (**C**) represents the total levels from the lipid and protein storages. The NO_2_-FAs values are the mean ± SEM of at least ten independent experiments. The statistical significance between means was analyzed by the Student’s *t*-test. Letter a indicates significant differences (*p* < 0.05) between the NO_2_-OA levels to the other two NO_2_-FAs in each developmental stage. This same statistical comparison was also made for NO_2_-LA and NO_2_-Ln. Significant differences (*p* < 0.05) are denoted by letters b and c, respectively. A comparison of each amount of NO_2_-FA in the different developmental stages was also made. Numbers 1, 2, 3, 4, 5, and 6 respectively indicate the significant differences (*p* < 0.05) between: the NO_2_-FAs levels in the seedlings and 5-day-old plants; the 5- and 14-day-old plants; the 14- and 24-day-old plants; the 24- and 34-day-old plants; the 34- and 36-day-old plants; and the 36- and 53-day-old plants.

After identifying the endogenous presence of NO_2_-OA, NO_2_-LA, and NO_2_-Ln in Arabidopsis, the NO_2_-FAs levels were quantified in different plant developmental stages. Figure 4 shows the endogenous levels of the NO_2_-FAs esterified with complex lipids (panel A), those which came from the adduction with proteins (panel B), and the total levels when considering both protein and lipid storages (panel C) in each plant development stage.

In the storage lipids, NO_2_-Ln was the most abundant, followed by NO_2_-OA and NO_2_-LA, both of which had a similar abundance. The NO_2_-FAs levels generally lowered during development, albeit with some fluctuations in certain developmental stages. Specifically in the stages when plants presented fully open cotyledons (5-day-old seedlings) and a mature rosette (24-day-old plants), the levels of the three NO_2_-FAs lowered (Figure 4A).

In the protein reservoir, the three NO_2_-FAs appeared at similar levels in the seed stage. However in the vegetative, generative, and senescence stages, NO_2_-LA was the major NO_2_-FA, followed by NO_2_-Ln and NO_2_-OA, and the levels of both were generally similar. When analyzing behavior on the whole, the NO_2_-FAs levels in seeds were a minority, but their content increased for the three NO_2_-FAs in the seedlings with open cotyledons (5-day-old seedlings). These high levels were similar during vegetative rosette growth (14- and 24-day-old plants) and during flowering (34-day-old plants). However, when plants initiated seed production (36 day-old plants), the NO_2_-FAs from the protein reservoir lowered, and these levels were similar in the senescent plants (53-day-old plants) (Figure 4B).

The compilation of previous data (Panels 4A and 4B) allowed us to establish that the total levels of the three NO_2_-FAs from the protein and lipid storages during development displayed similar behavior to that described above for the NO_2_-FAs esterified with complex lipids (Figure 4C).

### 3.4. Nitro-Fatty Acids Distribution in Storage Biomolecules during Arabidopsis thaliana Development

**Figure 5:**
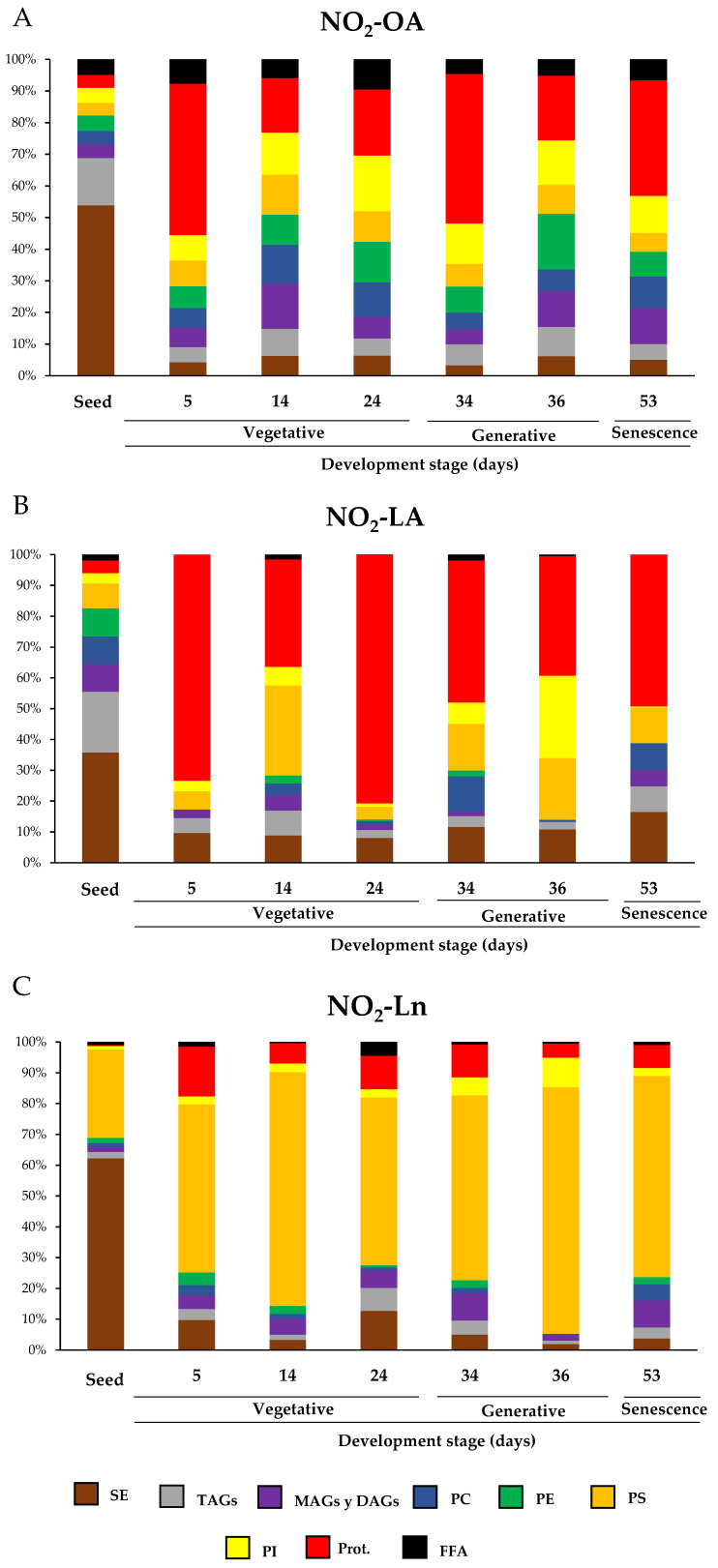
The relative distribution of NO_2_-FAs in the different storage biomolecules during Arabidopsis development. Graphs show NO_2_-FAs as the storage biomolecules (NO_2_-OA in panel (**A**); NO_2_-LA in panel (**B**); NO_2_-Ln in panel (**C**)) for each developmental stage and how this distribution is modulated during development. The seeds and plants corresponding to the vegetative (5-, 14-, and 24-day-old plants), generative (34- and 36-day-old plants) and senescence stages (53-day-old plants) were used to characterize Arabidopsis development. The 100% corresponds to the total content of each NO_2_-FA and its distribution in biomolecule storages in each of the development situations studied.

In this work, we identified the main NO_2_-FAs storage biomolecules implicated in the different analyzed growth stages. We selected different phases to cover the main events that take place during Arabidopsis development where the NO_2_-FAs levels in each reservoir were quantified. Figure 5 specifically shows the storage biomolecules of each NO_2_-FA in all the development stage. Hence, it indicates which storage biomolecule is the richest in NO_2_-OA (Panel A), NO_2_-LA (Panel B), or NO_2_-Ln (Panel C) in each development stage and how this abundance changes as plants grow and develop. Supplementary Table S1 displays the absolute values for the NO_2_-FAs levels for each reservoir biomolecule and development stage.

Our results showed that, during Arabidopsis development, the distribution of NO_2_-FAs in the lipid and protein storages was wide and ubiquitous. However, a detailed analysis of the NO_2_-FAs distribution in the different storage biomolecules evidenced for seeds that the three NO_2_-FAs were esterified mainly with SE, with levels of 0.366 ± 0.038 pmol/g FW for NO_2_-OA, 0.352 ± 0.072 pmol/g FW for NO_2_-LA, and 5.137 ± 1.002 pmol/g FW for NO_2_-Ln (Figure 5 and Supplementary Table S1). NO_2_-OA and NO_2_-LA were significantly stored in TAGs with absolute values of 0.101 ± 0.019 pmol/g FW and 0.194 ± 0.032 pmol/g FW, respectively, while PS (2.367 ± 0.329 pmol/g FW) was the preferential deposit for NO_2_-Ln and thus was the second NO_2_-FAs storage biomolecule in seeds (Figure 5 and Supplementary Table S1). In the vegetative stage, specifically when seed germination progressed (5-day-old seedlings), proteins were the main reservoir biomolecule of NO_2_-OA (0.1 ± 0.016 pmol/g FW), NO_2_-LA (0.2 ± 0.014 pmol/g FW), and, to a lesser extent, of NO_2_-Ln because the main storage for this NO_2_-FA was located in PS (0.479 ± 0.092 pmol/g FW) (Figure 5 and Supplementary Table S1). During rosette development, which occurs in the vegetative stage (14- and 24-day-old plants), phospholipids and proteins become the main storage biomolecules for NO_2_-OA (Figure 5). In contrast, NO_2_-LA and NO_2_-Ln showed the same abundance pattern as that described above; that is, they were essentially located in proteins and PS, respectively (Figure 5). Finally, in the generative (34- and 36-day-old plants) and senescence (53-day-old plants) stages, phospholipids and proteins were the most representative storage biomolecules for NO_2_-OA, proteins for NO_2_-LA, and PS for NO_2_-Ln (Figure 5).

In order to obtain information about NO_2_-FAs abundance in each storage biomolecule in all they development stages, we looked for the preponderant NO_2_-FA in each storage biomolecule in the different development stages.

**Figure 6:**
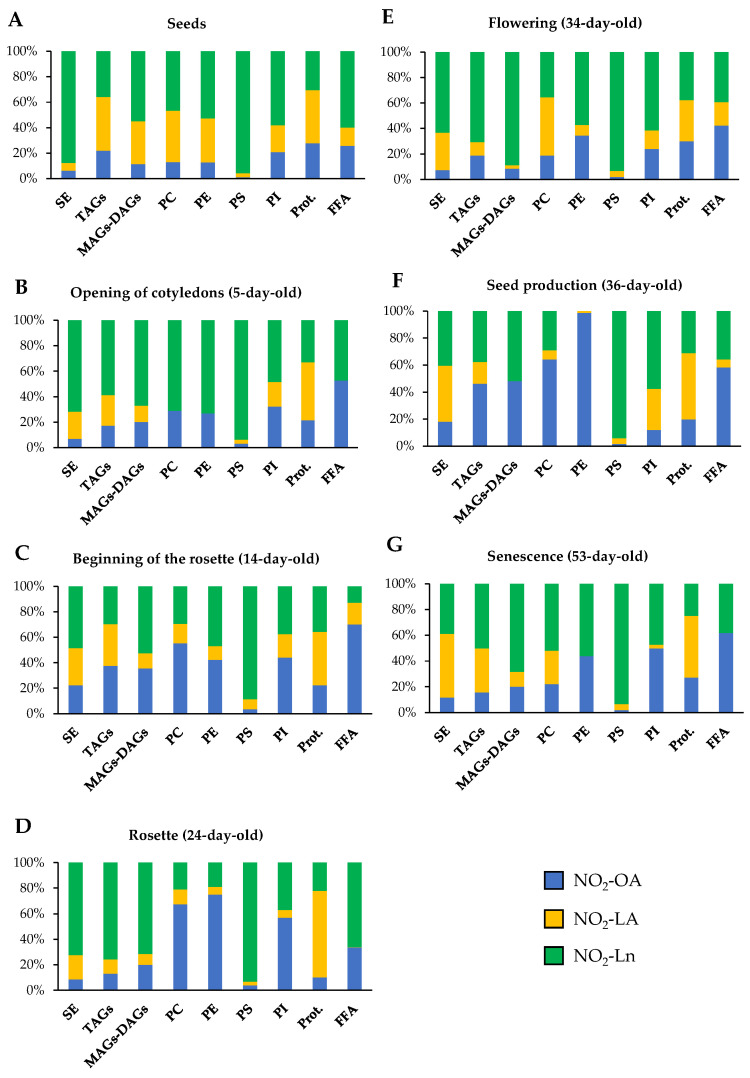
NO_2_-FAs relative abundance in the different storage biomolecules in each Arabidopsis development stage. This figure shows the relative concentration (expressed as a percentage) of each NO_2_-FA in the different lipid and protein storages present in seeds (**A**) and in the 5- (**B**), 14- (**C**), 24- (**D**), 34- (**E**), 36- (**F**), and 53-day-old plants (**G**). The 100% represents the sum of the total NO_2_-OA, NO_2_-LA, and NO_2_-Ln content in each of the biomolecules storages studied for each stage of development.

In the seed stage, NO_2_-OA presented relatively low abundance in each storage biomolecule compared to the other NO_2_-FAs, especially in PS. NO_2_-LA distribution was homogeneous except in the SE and PS reservoirs, where its presence was scarce. For NO_2_-Ln, its presence in SE and PS was predominantly noted although this NO_2_-FA was also found in the other storage biomolecules (Figure 6A and Supplementary Table S1). In the next growth stage, plants exhibited completely open cotyledons (5-day-old seedlings). In this case, the quantification of the MS/MS spectra showed that NO_2_-Ln was the predominant NO_2_-FA in most of the analyzed lipid storage biomolecules, which highlights its high esterification in PS. On the contrary, NO_2_-LA disappeared from the PC and PE deposits, and its contribution in the protein reservoir was marked. The participation of NO_2_-OA was homogeneous in the different storage biomolecules except for PS, where it was minimal (Figure 6B and Supplementary Table S1). In the 14-day-old seedlings, which phenotypically showed the onset of rosette formation, NO_2_-FAs were present in all the reservoirs, but their abundances differed. NO_2_-OA predominated in PC, NO_2_-LA in proteins, and PS remained as the main NO_2_-Ln storage biomolecule (Figure 6C and Supplementary Table S1). When plants presented a completely developed rosette (24-day-old plants), phospholipids accumulated mainly NO_2_-OA, which was not applicable to PS for being mostly esterified with NO_2_-Ln. NO_2_-LA was predominant in the protein deposit. NO_2_-Ln participation was greater in the other analyzed storage biomolecules (Figure 6D and Supplementary Table S1). The flowering 34-day-old seedlings played a predominant role for NO_2_-Ln, especially in PS and MAGs + DAGs. However, NO_2_-LA also played a major role in PC. NO_2_-OA was present in all the storage biomolecules but did not play a predominant role in any of them (Figure 6E and Supplementary Table S1). After flowering, the plant started seed production (36-day-old plants). In this phase, the dominance of NO_2_-OA in most storage biomolecules, especially in PC and PE, was noteworthy. For the other NO_2_-FAs, the abundance of NO_2_-Ln in PS and lack of NO_2_-LA in MAGs + DAGs were remarkable (Figure 6F and Supplementary Table S1). In the last plant development stage (senescence at 53 days), NO_2_-FAs presented a homogeneous abundance pattern, albeit with some exceptions: i.e., PE, where only NO_2_-OA and NO_2_-Ln were present; the observed MAGs + DAGs and PS reservoirs, where NO_2_-Ln almost completely dominated; and the quantities detected in PI where NO_2_-OA is highlighted (Figure 6G and Supplementary Table S1). 

In short, PC and PE were the main reservoir biomolecules for NO_2_-OA, proteins for NO_2_-LA, and TAGs and SE and PS for NO_2_-Ln.

The joint analysis of Figure 5 and Figure 6 showed that the SE storage in seeds was the main biomolecule for the three NO_2_-FAs (Figure 5), with NO_2_-Ln being the main one in this reservoir biomolecule (Figure 6A). In the vegetative stage, NO_2_-OA was distributed mostly in phospholipids and proteins (Figure 5). NO_2_-OA dominated only in the 14- and 24-day-old plants in PC and PE (Figure 6C,D). The distribution and abundance of NO_2_-LA were both located mostly in the protein storage (Figure 5 and Figure 6B–D). This same behavior was observed for NO_2_-Ln, but in this case, the main storage biomolecule was PS (Figure 5 and Figure 6B–D). Finally, in the generative and senescence stages, phospholipids and proteins continued to be the main NO_2_-OA reservoir biomolecules. However, there were some differences depending on the storage biomolecule and development stage. That was the case of the 36-day-old plants, where NO_2_-OA was exclusively detected in PC and PE, while NO_2_-LA and NO_2_-Ln presented the same behavior pattern as previously described in the vegetative stage (Figure 5 and Figure 6E–G).

## 4. Discussion

NO_2_-FAs are important signaling molecules that have been widely studied in animal systems. The actions mediated by NO_2_-OA, NO_2_-LA, NO_2_-cLA, and NO_2_-AA have been the focus of different works [4,5,6]. In plant systems, the first indications of the existence of NO_2_-FAs were obtained in olive fruit and EVOO, where the presence of NO_2_-cLA and cysteine-adducted-NO_2_-OA was specifically identified [8]. Currently, NO_2_-FAs have an ubiquitous distribution in plant organisms since the NO_2_-Ln endogenous presence has been identified in different plant species such as *Arabidopsis thaliana* [9], *Pisum sativum*, and *Oryza sativa* [10]. Moreover, NO_2_-OA has been recently described in *Brassica napus* plants [11]. The presence of NO_2_-FAs in plants is related to developmental signaling processes and stress responses [9,11]. Given the important signaling role of NO_2_-OA, NO_2_-LA, and NO_2_-Ln in both animal and plant systems, this work applied high-sensitivity technologies and detected the endogenous presence of NO_2_-OA and NO_2_-LA for the first time in *Arabidopsis thaliana*. In addition, the presence of the previously described NO_2_-Ln was confirmed (Figure 1). The quantification of the three NO_2_-FAs levels identified in Arabidopsis highlighted NO_2_-Ln as the most abundant, whereas NO_2_-LA and NO_2_-OA had lower but similar concentrations (Figure 2). These results are consistent with the availability of the corresponding unsaturated precursors in Arabidopsis, which may be susceptible to nitrate and might give rise to NO_2_-FAs. Thus, in the lipid profile of this plant species, Ln appeared as the majority in leaves and represents almost 50% of the total fatty acid content. The second most abundant was LA, followed by OA [66,67].

The addition of the nitro-group to unsaturated fatty acids converts NO_2_-FAs into potent electrophilic molecules that can establish adducts with proteins. In addition, NO_2_-FAs, like its non-nitrated forms, can also be esterified with complex lipids. Therefore, proteins and complex lipids can be considered NO_2_-FAs storage biomolecules [19,28]. In animal systems, a large group of proteins capable of binding to NO_2_-FAs has been identified [22,48,49,50,51,52,53,54,55,56]. In addition, TAGs have been identified as the main NO_2_-FAs storage lipid in these organisms [55]. However, the NO_2_-FAs storage biomolecules are completely unknown in plant systems. In this work, the MS/MS analyses performed on the 14-day-old seedlings, which represent a clear example of Arabidopsis vegetative growth, allowed NO_2_-FAs storage biomolecules to be identified in this plant species. Hence, the presence of NO_2_-OA, NO_2_-LA, and NO_2_-Ln bound to proteins and complex lipids, such as SE, TAGs, and different kinds of phospholipids, was identified (Figure 3 and Supplementary Figure S1).

The results obtained in this work revealed that phospholipids were the main reservoir of the three NO_2_-FAs. This was especially the case of NO_2_-Ln (Figure 3). Phospholipids are essential components of lipid bilayers for their amphipathic character [30,31]. Different types of phospholipids exist depending on the molecule to which the phosphate group is attached, such as PC, PE, PS, and PI. Based on our results, NO_2_-FAs were present in all the afore-mentioned phospholipid classes although they showed different abundance patterns. In general, PS were highlighted as the main NO_2_-FAs storage biomolecule, and this is especially applicable to NO_2_-Ln, whose esterification levels in PS were very high. For the other phospholipids, lower levels than those detected in PS were found but were similar for the three NO_2_-FAs (Supplementary Figure S1). Interestingly, the abundance of NO_2_-FAs in each phospholipid class did not correspond to their content in cell membranes. Indeed PC and PE were the main phospholipids in plant membranes, while PS and PI represented between 10% and 20% of the total phospholipid content [32,33].

Other albeit less representativeNO_2_-FAs storage biomolecules were MAGs, DAGs, and TAGs (Supplementary Figure S1). These molecules act mainly as a source of energy for young seedling growth, especially in early germination stages [46]. Furthermore, MAGs and DAGs are the common precursors of TAGs and membrane lipids (phospholipids). In leaves, most DAGs participate in the synthesis of membrane lipids that will be used for cell growth and proliferation and also for the maintenance of membranes [47]. SE were the most residual NO_2_-FAs storage lipids in juvenile plants (14-day-old) (Supplementary Figure S1). SE involve the esterification of a fatty acid with phytosterols, such as β-sitosterol, stigmasterol, campesterol, and cholesterol [44]. These molecules are found in plasma membranes and are implicated in the maintenance of the homeostasis of the phytosterols in them because, together with phospholipids and sphingolipids, they are essential plasma membrane components [36].

In addition to NO_2_-FAs lipid storages, NO_2_-FAs can also appear adducted with proteins. This makes them an important source of NO_2_-FAs because the levels detected in proteins were similar to those quantified in TAGs in the 14-day-old Arabidopsis plants (Supplementary Figure S1).

In summary, NO_2_-FAs are mainly localized in biomembranes, where they are esterified with phospholipids and SE. The presence of NO_2_-FAs in biomembranes can modulate physical bilayer properties and can even indirectly influence transmembrane proteins. In a recent study, the effect of the presence of NO_2_-FAs in biomembranes was evaluated by treating a model membrane with NO_2_-OA. The researchers found that NO_2_-FA altered the organization of membrane lipids, which led to not only lipid bilayer reorganization but also to the modulation of the structure and function of membrane-associated proteins [29]. The marked presence of NO_2_-FAs is also highlighted, especially NO_2_-Ln in PS. This phospholipid kind is considered a signaling lipid that regulates membrane-associated processes [35]. Therefore, the presence of NO_2_-FAs in PS could implicate them in these signaling processes.

After identifying the main NO_2_-FAs storage biomolecules in *Arabidopsis thaliana*, a study of the distribution of the NO_2_-FAs inside them during plant development was carried out. The starting point for plant development is seeds. The highest total NO_2_-OA, NO_2_-LA, and NO_2_-Ln levels were found in this plant material type (Figure 4C and Supplementary Table S1). However, when the NO_2_-FAs levels stored in lipid and protein reservoirs were analyzed separately, opposite behaviors were observed. High levels of complex lipid-esterified NO_2_-FAs and lesser abundance of protein-adducted NO_2_-FAs were quantified (Figure 4A,B). This NO_2_-FAs storage pattern was expected because seeds contain more than 60% lipids but also contain carbohydrates and proteins in smaller proportions [68]. The most abundant lipids in seeds are TAGs and SE, and both storage biomolecules are mobilized during germination to facilitate seedling growth in early stages [69]. In our study, these lipids (TAGs and SE), together with PS, contained the majority of NO_2_-FAs. Specifically, 50% NO_2_-OA, 35% NO_2_-LA, and 60% NO_2_-Ln were found to be esterified with SE, which proved to be the main NO_2_-FAs storage biomolecule in seeds. The contents of NO_2_-OA (15%) and NO_2_-LA (20%) in TAGs and the abundance of NO_2_-Ln (30%) in PS were also noteworthy (Figure 5 and Supplementary Table S1). Therefore, generally in seeds, NO_2_-FAs were mainly esterified with lipids with an energy function (TAGs) by acting as a source of sterols for membrane generation (SE) or by forming part of membranes (PS). In addition, the NO_2_-FAs esterified with complex lipids in seeds could act as stable NO reservoirs with the ability to donate NO to favor the germination process. In line with this, it has been reported that ABI5 transcription factor degradation by S-nitrosylation initiates seed germination [70].

Next, the Arabidopsis development stages consisting of seed germination and vegetative plant growth were studied. For this purpose, the newly germinated seedlings showing a radicle, a hypocotyl, and two completely open cotyledons (at 5 days), along with the seedlings in which rosette arrangement began to be noted (at 14 days) and the plants displaying a mature rosette (at 24 days), were analyzed. The total NO_2_-FAs levels progressively lowered, which was mainly motivated by the reduction of NO_2_-FAs esterification with complex lipids. However, adduction with proteins increased in these stages (Figure 4). The decrease observed in NO_2_-FAs content in the lipid storage, especially in relation to TAGs and SE (Figure 5), can be associated with the hydrolysis of TAGs to FFA and glycerol by the action of lipase enzymes [71]. Glycerol can be phosphorylated to undergo gluconeogenesis after its conversion into dihydroxyacetone phosphate (DHAP) [72]. FFA can be transported to the peroxisome, where they are activated to acyl-CoA to initiate β-oxidation. The β-oxidation product, acetyl-CoA, enters the glyoxylate cycle and, via the gluconeogenic pathway, provides the glucose required by the embryo as a source of energy during germination [73,74]. The scientific literature also includes SE mobilization processes during membrane development [69]. Something similar occurs with DAGs, which are precursor molecules of both TAGs and phospholipids, as indicated above. During germination and vegetative growth, most DAGs are used for membrane lipid assembly in areas where cell biogenesis, membrane expansion, and maintenance take place [47]. Consequently, significant amounts of TAGs do not accumulate in these plant growth phases [46].

Phospholipases, which are capable of hydrolyzing several types of phospholipid bonds, also play an important role in the vegetative stage [75]. Specifically, phospholipases act as key regulators during membrane organization by remodeling their lipids component, including NO_2_-FA. Consequently, they influence the physical properties of membranes [76]. By way of example, it has been described that phospholipase A1 (PLA1), which is expressed at high levels in juvenile plants, hydrolyses the acyl group at the sn-1 position of phospholipids and produces FFA and lysophospholipids. PLA1 shows increased expression during young rosette development [77]. Moreover, the scientific literature points out that these enzymes catalyze the release of Ln, which is the precursor of jasmonic acid, an organic compound involved in plant growth and development [78]. Another protein type that hydrolyses phospholipids, specifically at the sn-2 position, is phospholipase A2 (PLA2), and it also interacts with auxins. In particular, PLA2 and the product of its activity, lysophosphatidic ethanolamine, are required for the trafficking of PINs (auxin exit transporters) to the plasma membrane. This interaction is necessary for proper root growth [79] and correct endomembrane system organization during development and germination processes [80,81].

Generally, during seed germination and in the vegetative stage, phospholipids, as the main constituents of membranes, and proteins play a major role in plant growth [46]. They are also the main NO_2_-FAs storage biomolecules. Specifically, in this work, the distribution of NO_2_-OA was homogeneous between phospholipids and proteins, and NO_2_-LA was stored mostly in proteins, which associated both NO_2_-FAs with biological signaling processes. In contrast, NO_2_-Ln abundance in phospholipids was remarkable, especially in PS (Figure 5). At this point, it is important to mention that plant cell membranes are composed mainly of three lipid classes, namely glycerolipids, sphingolipids, and sterols, of which glycerolipids are the most abundant [33]. Glycerolipids are divided, in turn, into galactolipids, sulfolipids, TAGs, and phospholipids. The last are the most abundant in membranes [82]. PC and PE are the major phospholipids in membranes and are often considered “structural” lipids because they are essential for establishing a hydrophobic barrier in the membrane. In contrast, PS and PI are less abundant in membranes and act as “signaling” molecules because they mostly have regulatory effects on membrane functions. As these signaling lipids are negatively charged, they can interact with specific proteins and regulate almost all membrane-associated events [34,35,83]. Hence, the presence of NO_2_-FAs in different membrane lipid components during germination and in the vegetative stage was analyzed. Our results showed that approximately more than 30% NO_2_-OA, 10% NO_2_-LA and, above all, more than 70% NO_2_-Ln were esterified with some phospholipid type (Figure 5). Although very little is known about the role of NO_2_-FAs in biomembranes, different computer simulation techniques have shown that NO_2_-OA can form domains that cause membrane reorganization with the concomitant modification of the dynamic structure of their integral proteins [29]. In particular, the presence of NO_2_-Ln in a signaling complex lipid such as PS (Figure 5) could be related to its involvement in some processes, such as cell signaling, molecular trafficking, and cell division and growth. All these possibilities are supported by the presence of the nitro-group, which makes NO_2_-Ln a very reactive molecule that can carry out the PTM of proteins by nitroalkylation [7,84] or can act as a NO donor by giving rise to the S-nitrosylation PTM [15,17].

In short, NO_2_-FAs levels are lowered in the vegetative stage, especially in the TAGs and SE storages. This behavior could be justified by TAGs degradation to obtain energy and SE hydrolysis to generate the membranes needed to develop photosynthetic structures, root systems, stems, and leaves. In these growth stages, the remodeling of NO_2_-FAs storage biomolecules also occurs because, although TAGs and SE are major reservoirs in seeds, they are minor ones during seed germination and in the vegetative stage when phospholipids and proteins acquire a more relevant role and confer NO_2_-FAs functional and signaling implications.

Once the plant presents a complete mature rosette (24-day-old plants), it starts the generative stage with flowering. It is noteworthy that the NO_2_-FAs levels stored in PC were lower in plants with a complete rosette (at 24 days) in relation to the plants displaying a juvenile rosette (14-day-old seedlings). Specifically a 72% decrease was detected for NO_2_-OA, one of 81% for NO_2_-LA, and one of 83% for NO_2_-Ln (Supplementary Table S1). The diminished presence of NO_2_-FAs in PC, which was more pronounced in NO_2_-Ln in the plants whose rosette development had been completed, could be related to the next flowering step because such a process is initiated by the transcription of the FLOWERING LOCUS T (FT) gene, which gives rise to a mobile protein that induces flowering in plants [85]. Despite PC being a “structural” lipid, it participates in the flowering process because different works conducted with mutant plants have established that seedlings with high PC levels show early flowering, while the opposite occurs in plants with lower PC contents [86,87]. Interestingly, the flowering phenotype observed in the plants with higher PC contents depended on the expression of FT, which was directly influenced by the high saturated fatty acids composition of PC. The saturated or unsaturated fatty acids content in PC is influenced by photoperiod, with saturated fatty acids predominating in the daytime and unsaturated content at night [86,88]. Therefore, the punctual decrease in the NO_2_-FAs content in PC detected in the 24-day-old plants could modulate the expression of the gene to favor flowering (FT). This decrease was restricted to that phase because in those plants, in a more advanced flowering stage with open flowers (34-day-old plants), the NO_2_-FAs levels in PC recovered, whose values were similar to those detected in the seedlings with a juvenile rosette (14-day-old seedlings) (Supplementary Table S1).

Next, the plant enters a generative period when it spends its energy on reproduction by developing flowers (34-day-old plants) from which fruit and seeds are generated (36-day-old plants). In this stage, the main NO_2_-FAs storage biomolecules continued to be proteins and phospholipids (Figure 5). The marked presence of NO_2_-Ln in PS should be particularly underlined (Figure 6), which is a phospholipid that is relevant in reproductive organ formation. Specifically, different studies performed in Arabidopsis plants deficient in phosphatidylserine synthase 1 (PSS1), a key protein in the synthesis of PS from PE via a base exchange mechanism, have demonstrated a reduction in the inflorescence meristem and evidenced plants’ inability to develop new floral meristems. Therefore, the presence of PSS1 and thus PS plays an essential role in floral meristem maintenance and development through the activation of the CLAVATA (CLV)-WUSCHEL (WUS) signaling pathway [89,90]. These results suggest the possible involvement of NO_2_-FAs to signal flowering initiation and also in the maintenance and development of secondary floral structures.

Furthermore, in the generative stage, specifically during flowering, phospholipids and proteins continued to be the main NO_2_-FAs storage biomolecules. However, when the plant obtained its first fruit, which are called siliques in Arabidopsis, the total NO_2_-FAs levels slightly lowered in both the lipid and protein reservoirs although the reduction in the latter was more pronounced (Figure 4). This behavior could be associated with the beginning of the senescence stage because the 36-day-old plants showed mature siliques and represented an intermediate stage between the generative and senescence stages. When senescence starts, plants enter a state of programmed cell death because photosynthetic capacity reduces when chloroplast thylakoid membranes are degraded. These membranes are formed by glycolipids, and an increase in fatty acids release occurs as a consequence of glycolipid degradation. These processes cause TAGs to accumulate in senescent leaves [91,92]. The phospholipids and sterols located in extraplasmatic membranes, such as the plasma membrane, tonoplast, and endoplasmic reticulum, also decrease during senescence. Phospholipase activity increases in phospholipids, which leads to membrane degradation, and in the case of sterols, senescence generates SE accumulation [93,94]. Briefly, senescence brings about loss of plants’ photosynthetic capacity because chloroplasts’ thylakoid membranes degrade, and chlorophyll levels decrease. Extraplasmatic membranes also degrade, which reduces membrane fluidity. All this leads to the mobilization of nutrients from leaves, which causes lipids accumulation in seeds, such as TAGs and SE [94,95].

The last stage development to be characterized was senescence. To study this phase, we used plants with advanced senescence (at 53 days), in which very low NO_2_-FAs levels were quantified in both the lipid and protein deposits (Figure 4). The drop in NO_2_-FAs levels could be a consequence of membrane phospholipids hydrolysis because it was in these complex lipids where NO_2_-FAs were localized mainly in earlier growth stages. It is well-established in the senescence stage that ROS levels increase [80] and can cause the oxidation of the Michael adducts generated between proteins and NO_2_-FAs, which would lead to NO_2_-FA release [20,48]. All these mechanisms would cause NO_2_-FAs release from the membranous structures present in leaves and their mobilization towards seeds, where they would be integrated into lipids that can act as a source of either energy (TAGs) or sterols for membrane generation (SE). All this would explain the high NO_2_-FAs levels quantified in seeds, to which the possible nitration of unsaturated fatty acids as a consequence of the high oxidative state in senescent cells would contribute.

Finally, it is worth mentioning that the FFA levels in each development stage were low (Figure 5 and Figure 6). This low abundance could be motivated by their high reactivity, which would justify their quick esterification with lipids or their adduction with proteins [19,28].

## 5. Conclusions

**Figure 7:**
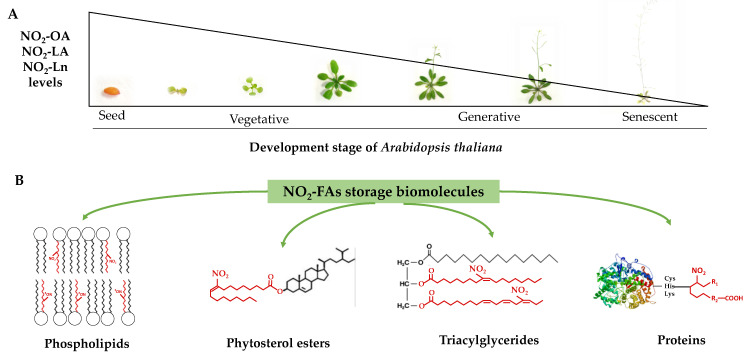
The NO_2_-OA, NO_2_-LA, and NO_2_-Ln levels during *Arabidopsis thaliana* development (panel (**A)**) and the identification of phospholipids, phytosterol esters, triacylglycerides, and proteins as NO_2_-FAs storage biomolecules in Arabidopsis (panel (**B**)).

In this work, the presence of nitro-oleic acid and nitro-linoleic acid in *Arabidopsis thaliana* was identified and quantified for the first time. Additionally, the presence of nitro-linolenic acid in this plant species was corroborated, which played a predominant role in the analyzed processes (Figure 7). The characterization of the distribution of nitrated fatty acids at the cellular level, unknown to date, allowed the establishment of phytosterol esters; mono-, di-, and triacylglycerides; phosphatidylcholine, phosphatidylethanolamine, phosphatidylserine, and phosphatidylinositol as the main lipid reservoirs of these molecules. Our results reflect a heterogeneous distribution of nitro-fatty acids in cellular compartments and their preferential localization in lipid storage upon the protein reservoir (Figure 7). 

The study carried out throughout Arabidopsis development showed a decreasing pattern in the nitro-fatty acids content stored in both the protein and lipid reservoirs (Figure 7). Phytosterol esters and triacylglycerides were the preferential lipid storages in seeds, which were replaced with phospholipids and proteins in the vegetative, reproductive, and senescence stages. The majority esterification of the nitro-fatty acids in phospholipids, specifically in phosphatidylserine, suggested that nitro-fatty acids were involved in biomembrane dynamics by directly influencing their biophysical properties and also in phosphatidylserine-mediated signaling phenomena. The decreased content of nitro-fatty acids in phosphatidylcholine would limit the inhibitory effect exerted by unsaturated fatty acids on the flowering process initiation. 

Therefore, this work provides a detailed study of the role of nitro-fatty acids during plant development by highlighting the possible implications of these molecules in decisive events for plant development. It also opens up a new research field to analyze and study the possible role and interactions between nitro-fatty acids and phospholipids and with the proteins in the signaling processes that occur during plant development.

## Data Availability

Data are contained within the article and Supplementary Material.

## Supplementary Materials

The following supporting information can be downloaded at: https://www.mdpi.com/article/10.3390/antiox11101869/s1, Figure S1: Distribution and quantification of the endogenous levels of NO2-FAs in the different storage biomolecules; Table S1: The endogenous levels of NO2-OA, NO2-LA, and NO2-Ln in the different storage biomolecules in the selected Arabidopsis thaliana development stages.
