# Supplementary material for: Nitrated Fatty-Acids Distribution in Storage Biomolecules during Arabidopsis thaliana Development
Source: Antioxidants (Basel). 2022 Sep 21;11(10):1869. doi: 10.3390/antiox11101869 (PMC9598412; doi:10.3390/antiox11101869)
Supplement: Supplementary file 1 [file antioxidants-11-01869-s001.zip › antioxidants-1897410-supplementary.pdf]

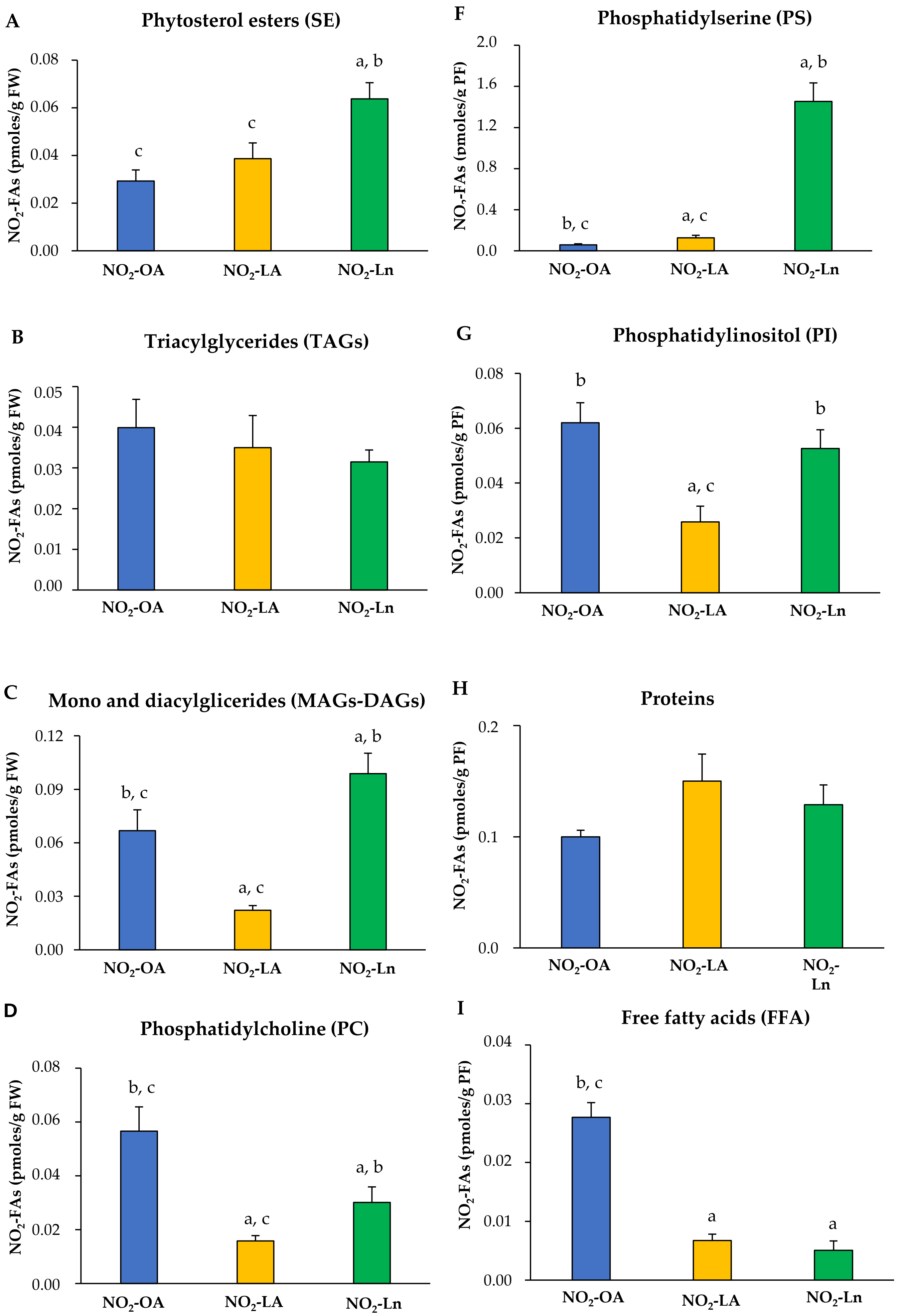

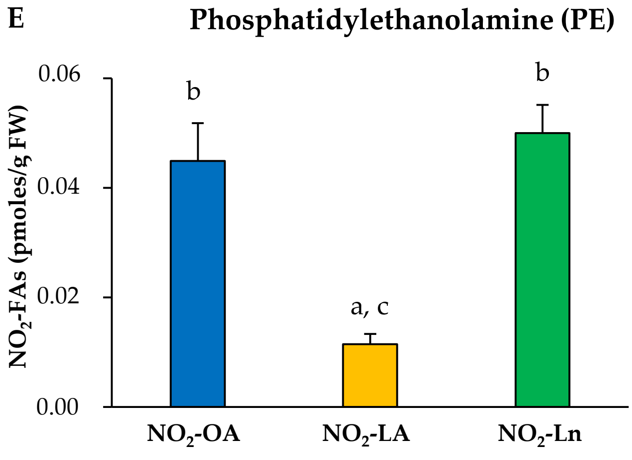


**Supplementary Figure S1.** Distribution and quantification of the endogenous levels of NO$_2$-FAs in the different storage biomolecules. The figure shows the levels of NO$_2$-OA, NO$_2$-LA and NO$_2$-Ln found in the different biological stores, such as SE (A), TAGs (B), MAGs-DAGs (C), PC (D), PE (E), PS (F), PI (G), proteins (H), as well as the FFA levels (I) identified in the 14-day-old Arabidopsis seedlings. The NO$_2$-FAs values are the mean±SEM of at least ten independent experiments. The statistical significance between means was analysed by the Student's t-test. Letters a, b and c indicate significant differences ($p<0.05$) in relation to NO$_2$-OA, NO$_2$-LA and NO$_2$–Ln, respectively.

**Supplementary table S1.** The endogenous levels of $NO_2$-OA, $NO_2$-LA and $NO_2$-Ln in the different storage biomolecules in the selected *Arabidopsis thaliana* development stages.

| Stage | Storage | $NO_2$-OA (pmoles/g FW) | $NO_2$-LA (pmoles/g PF) | $NO_2$-Ln (pmoles/g PF) |
|---|---|---|---|---|
| Seed 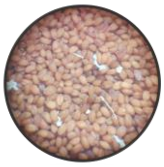 | SE | 0.366±0.038 | 0.352±0.072 | 5.137±1.002 |
| | TAGs | 0.101±0.019 | 0.194±0.032 | 0.165±0.028 |
| | MAGs+DAGs | 0.029±0.004 | 0.087±0.007 | 0.142±0.028 |
| | PC | 0.029±0.004 | 0.089±0.012 | 0.103±0.013 |
| | PE | 0.033±0.004 | 0.090±0.023 | 0.137±0.032 |
| | PS | 0.027±0.003 | 0.079±0.010 | 2.367±0.329 |
| | PI | 0.032±0.003 | 0.033±0.006 | 0.091±0.011 |
| | Proteins | 0.030±0.011 | 0.040±0.014 | 0.030±0.013 |
| | FFA | 0.033±0.005 | 0.018±0.002 | 0.077±0.012 |
| Opening of cotyledons 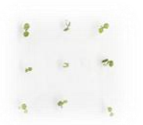 (5-day-old) | SE | 0.008±0.001 | 0.026±0.002 | 0.086±0.009 |
| | TAGs | 0.009±0.001 | 0.013±0.002 | 0.031±0.001 |
| | MAGs+DAGs | 0.012±0.001 | 0.007±0.002 | 0.039±0.006 |
| | PC | 0.012±0.001 | 0.012±0.001 | 0.028±0.005 |
| | PE | 0.013±0.002 | ND | 0,036±0,007 |
| | PS | 0.016±0.002 | 0.016±0.004 | 0.479±0.092 |
| | PI | 0.015±0.002 | 0.009±0.001 | 0.023±0.001 |
| | Proteins | 0.100±0.016 | 0.200±0.014 | 0.140±0.025 |
| | FFA | 0.015±0.001 | ND | 0.013±0.001 |
| Beginning of the rosette 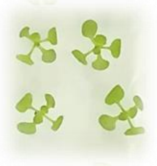 | SE | 0.029±0.005 | 0.039±0.007 | 0.064±0.022 |
| | TAGs | 0.040±0.007 | 0.035±0.008 | 0.032±0.004 |
| | MAGs+DAGs | 0.067±0.012 | 0.022±0.003 | 0.099±0.043 |
| | PC | 0.057±0.009 | 0.016±0.002 | 0.030±0.010 |
| | PE | 0.045±0.007 | 0.011±0.002 | 0.050±0.014 |
| | PS | 0.059±0.010 | 0.127±0.024 | 1.453±0.165 |
| | PI | 0.062±0.007 | 0.026±0.006 | 0.053±0.002 |
| | Proteins | 0.100±0.006 | 0.150±0.024 | 0,130±0,018 |
| | FFA | 0.028±0.002 | 0.007±0.001 | 0.005±0.001 |
| Rosette (24-day-old) 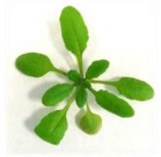 | SE | 0.009±0.001 | 0.020±0.004 | 0.077±0.007 |
| | TAGs | 0.008±0.001 | 0.007±0.001 | 0.045±0.008 |
| | MAGs+DAGs | 0.010±0.001 | 0.004±0.002 | 0.035±0.006 |
| | PC | 0.016±0.002 | 0.003±0.001 | 0.005±0.002 |
| | PE | 0.019±0.002 | 0.002±0.001 | 0.005±0.001 |
| | PS | 0.014±0.004 | 0.010±0.001 | 0.329±0.034 |
| | PI | 0.025±0.003 | 0.003±0.001 | 0.017±0.003 |
| | Proteins | 0.030±0.003 | 0.200±0.030 | 0.070±0.023 |
| | FFA | 0.014±0.003 | ND | 0.027±0.002 |
| Flowering (34-day-old) 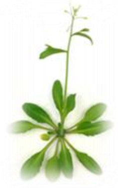 | SE | 0.009±0.002 | 0.036±0.006 | 0.077±0.016 |
| | TAGs | 0.019±0.003 | 0.011±0.002 | 0.070±0.008 |
| | MAGs+DAGs | 0.013±0.002 | 0.004±0.002 | 0.137±0.025 |
| | PC | 0.015±0.002 | 0.036±0.008 | 0.028±0.005 |
| | PE | 0.023±0.004 | 0.005±0.002 | 0.038±0.007 |
| | PS | 0.020±0.004 | 0.046±0.002 | 0.928±0.094 |
| | PI | 0.035±0.007 | 0.021±0.007 | 0.090±0.014 |
| | Proteins | 0.130±0.036 | 0.140±0.066 | 0.160±0.059 |
| | FFA | 0.013±0.002 | 0.006±0.001 | 0.012±0.001 |

| | | | | |
|---|---|---|---|---|
| Seed production | SE | 0.011±0.002 | 0.024±0.003 | 0.024±0.006 |
| | TAGs | 0.016±0.001 | 0.006±0.001 | 0.013±0.001 |
| 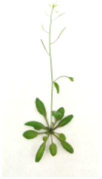 | MAGs+DAGs | 0.019±0.001 | ND | 0.021±0.004 |
| | PC | 0.012±0.002 | 0.001±0.000 | 0.005±0.003 |
| | PE | 0.030±0.005 | ND | ND |
| | PS | 0.016±0.002 | 0.044±0.004 | 0.947±0.097 |
| | PI | 0.024±0.004 | 0.060±0.011 | 0.113±0.023 |
| | Proteins | 0.040±0.005 | 0.090±0.029 | 0.060±0.025 |
| (36-day-old) | FFA | 0.009±0.001 | 0.001±0.001 | 0.005±0.003 |
| Senescence | SE | 0.005±0.001 | 0.021±0.003 | 0.016±0.002 |
| | TAGs | 0.005±0.001 | 0.011±0.001 | 0.015±0.002 |
| 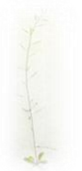 | MAGs+DAGs | 0.011±0.002 | 0.006±0.003 | 0.038±0.006 |
| | PC | 0.010±0.001 | 0.011±0.002 | 0.022±0.003 |
| | PE | 0.008±0.001 | ND | 0.010±0.002 |
| | PS | 0.006±0.001 | 0.014±0.002 | 0.280±0.035 |
| | PI | 0.001±0.002 | 0.001±0.001 | 0.011±0.002 |
| | Proteins | 0.040±0.009 | 0.060±0.008 | 0.030±0.002 |
| (53-day-old) | FFA | 0.006±0.001 | ND | 0.004±0.002 |

ND: not detected.
